# Sorption of CO_2_ and CH_4_ on Raw and Calcined Halloysite—Structural and Pore Characterization Study

**DOI:** 10.3390/ma13040917

**Published:** 2020-02-19

**Authors:** Anna Pajdak, Norbert Skoczylas, Arkadiusz Szymanek, Marcin Lutyński, Piotr Sakiewicz

**Affiliations:** 1 Strata Mechanics Research Institute of the Polish Academy of Sciences, 27 Reymonta St., 30-059 Krakow, Poland; skoczylas@imgpan.pl; 2 Faculty of Mechanical Engineering and Computer Science, Czestochowa University of Technology, 21 Armii Krajowej Av, 42-200 Czestochowa, Poland; szymanek@imc.pcz.czest.pl; 3 Department of Mining, Faculty of Mining, Safety Engineering and Industrial Automation, Silesian University of Technology, 2 Akademicka St., 44-100 Gliwice, Poland; marcin.lutynski@polsl.pl; 4 Faculty of Mechanical Engineering, Institute of Engineering Materials and Biomaterials, Division of Nanocrystalline and Functional Materials and Sustainable Proecological Technologies, Silesian University of Technology, 18a Konarskiego St., 44-100 Gliwice, Poland; piotr.sakiewicz@polsl.pl

**Keywords:** halloysite, pore structure, sorption capacity of CH_4_ and CO_2_, kinetics of diffusion, effective diffusion coefficient

## Abstract

The article presents comparative characteristics of the pore structure and sorption properties of raw halloysite (R-HAL) and after calcination (C-HAL) at the temperature of 873 K. Structural parameters were determined by optical scanning and transmission electron microscopy methods as well as by mercury porosimetry (MIP, Hg) and low-pressure nitrogen adsorption (LPNA, N_2_, 77 K). The surface area parameter (LPNA) of halloysite mesopores before calcination was 54–61 m^2^/g. Calcining caused the pore surface to develop to 70–73 m^2^/g. The porosity (MIP) of halloysite after calcination increased from 29% to 46%, while the surface area within macropores increased from 43 m^2^/g to 54 m^2^/g. The total pore volume within mesopores and macropores increased almost twice after calcination. The course of CH_4_ and CO_2_ sorption on the halloysite was examined and sorption isotherms (0–1.5 MPa, 313 K) were determined by gravimetric method. The values of equilibrium sorption capacities increased at higher pressures. The sorption capacity of CH_4_ in R-HAL was 0.18 mmol/g, while in C-HAL 0.21 mmol/g. CO_2_ sorption capacities were 0.54 mmol/g and 0.63 mmol/g, respectively. Halloysite had a very high rate of sorption equilibrium. The values of the effective diffusion coefficient for methane on the tested halloysite were higher than *De* > 4.2 × 10^−7^ cm^2^/s while for carbon dioxide *De* > 3.1 × 10^−7^ cm^2^/s.

## 1. Introduction

Coal has been one of the dominant fuels of the world and European economy since the industrial revolution in the XIXth century. In 1990, coal made up almost 41% of gross energy consumption in current EU-28 Member States and 39% of energy production in these countries. Despite the ‘climate package’ and decarbonisation policy, in 2015 16% of the EU’s gross energy consumption came from coal and 24% came from electricity production [1]. According to global policy, the use of coal as a fuel is to cease so solutions are sought to use this raw material and its deposits in an alternative way, e.g., as reservoirs for carbon dioxide carbon capture and storage (CCS) technology. In CCS and carbon capture and utilization (CCU) technologies, CO_2_ is captured from flue gas and either transported to a storage site (CCS) or used for other purposes such as chemical conversion to methane [2,3,4,5]. Coal can also use enhanced coal bed methane recovery (ECBM) technology, which includes CO_2_ storage and simultaneous acquisition of CH_4_ for energy purposes [6,7]. Over the years, CO_2_ capture technologies such as CCS and ECBM have been studied under laboratory conditions [8,9,10,11,12] and modeled [13,14]. However, this technology has still not obtained sufficient social acceptance to be implemented on a global scale.

Coal still remains a major fossil fuel in the world economy, accounting for 27% of all energy used worldwide and making up 38% of electricity generation [15]. Its properties and conversion are the subject of many studies [16,17,18]. Its conversion is a source of many pollutants, in addition to CO_2_, also acid type pollutants (NO_x_, SO_2_, HCl, HF) and mercury [19,20]. Neutralization of these pollutants has been an object of interest for many years [21,22,23,24,25]. The most common and advanced CO_2_ capture technology from flue gas arising during the production of energy electricity from coal is absorption using amines, mainly monoethylamine (MEA). This technology, however, is a very energy-intensive process not only from the point of view of operation, but also solvent production. The environmental impact of this process is very large when taking into account solvent production, use and regeneration. The production of MEA from ammonia involves important CO_2_ emissions during the Haber–Bosch process [26,27]. The regeneration of the solvent after the absorption is also an indirect source of CO_2_ related to the use of fuels. Other drawbacks of this technology include a high rate of equipment corrosion and loss of solvent due to evaporation during the process [28]. The regeneration of MEA absorbent is energy consuming. Overall, the energy consumption of the MEA process is approximately 3.8 MJ/kg of CO_2_ captured [29]. The alternative solution is the adsorption of CO_2_ on solid sorbents with the use of e.g., the pressure swing adsorption (PSA) method or the temperature swing adsorption (TSA) method. In the case of PSA, the amount of energy required is slightly lower i.e., 1.2–3.2 MJ/kg of CO_2_ captured depending on the process [30,31]. PSA technology is mainly used to remove CO_2_ from synthesis gas for hydrogen production, but it can also be used to remove CO_2_ in post-combustion and pre-combustion processes [31]. When considering adsorption on solid sorbents the following criteria for sorbents have to be met: high CO_2_ sorption capacity, CO_2_ adsorption selectivity in relation to other gases in the mixture (particularly N_2_ in the case of flue gas), high regeneration efficiency, availability, thermal, chemical and mechanical stability, low cost of adsorbent and low environmental footprint of sorbent production or modification. In general, the following groups of solid sorbents for PSA or TSA technologies can be distinguished [5,27,29,32,33]: zeolites, activated carbonaceous materials, metal oxides, silicas, hydrotalcite-like supports, metal-organic frameworks (MOFs), polymers, clay minerals.

The least explored group of materials as potential CO_2_ adsorbents are clay minerals. They are used mostly for liquid absorption and there is little knowledge about the adsorption of CO_2_/CH_4_ mixtures on clay minerals, particularly with reference to the capture methods. One of the clay minerals which exhibits good sorption properties is halloysite. There have been attempts to investigate CO_2_ adsorption on halloysite nanotubes [34], or pure halloysite minerals at moderate pressure [35]. Research into other amine modified minerals, such as amorphous clay minerals or amino acid/acid modified montmorillonite clays, shows that this group of sorbents may be potentially interesting for PSA technology [36,37,38]. Table 1 shows a summary of sorption capacities of various solid sorbents used for adsorption of CO_2_ and CH_4_. It is clear that carbon based adsorbents, MOFs or zeolites, have much higher sorption capacity. Nevertheless, one must have in mind the costs, particularly for applications where large amounts of adsorbent need to be used. In the case of clay minerals the unit cost can be as much as 100 times lower in comparison to MOFs, silicas or synthetic zeolites [36]. Modification costs for clay minerals are much lower and liquid modifiers used are usually environmentally friendly.

The article presents a study of CO_2_/CH_4_ high pressure sorption calcined and raw halloysite. An in-depth analysis of pore structure was performed to evaluate halloysite for further structural modifications.

## 2. Methods

The microscopic analyses of the halloysite surface topography were carried out using an Apollo XP scanning electron microscope (SEM-EDS, EDAX, Weiterstadt, Germany). The microscope consists of an EDAX Apollo X energy-dispersive X-ray spectrometer with a secondary electron and back scattered electron detectors and an EDS X-ray dispersion energy spectrometer, on which, in the micro-areas of the sample, the chemical composition of the rocks has been identified. The measurements were carried out in a low-vacuum mode (10^−2^ Pa) with an accelerating voltage of 20 kV electron beam. The tests were carried out for halloysite samples before and after calcining. Before measuring, the samples were sprayed with a thin layer of silver. Images were recorded at magnifications of 100–2500×. The Apollo XP scanning electron microscope is owned by the Academic Centre for Materials and Nanotechnology, AGH University of Science and Technology in Poland.

For further characterization of the synthesised halloysite specimens, transmission electron microscope (TEM) studies were performed. Before observations of the halloysite nanoplates and nanotubes, research material was prepared. A small amount of halloysite sample in ethanol was dispersed in ethanol and a drop of suspension was placed on a microscopic carbon-coated copper mesh. TEM tests were carried out using a Titan 80–300 TEM/STEM emission electron microscope (FEI, Hillsboro, OR, USA) with emission transmission equipped with a twin-lens operated at 300 kV and an annular darkfield detector.

The structural analyses of the surface area and the micropore and mesopore size distribution were carried out by low pressure nitrogen adsorption (LPNA) on a ASAP 2020 analyser (Micromeritics, Norcross, GA, USA). For the measurements nitrogen was used as the adsorbate. The analyses were carried out under isothermal conditions at 77 K and in the absolute pressure range of 0–0.1 MPa. Such a pressure range corresponds to the relative pressure that is equal to the ratio of the absolute pressure and the critical pressure of N_2_ in the gas phase, in the range of 0 < *p*/*p*_0_ < 0.996. Prior to the analyses the samples were degassed at 343 K for 24 h in pressure close to vacuum. The measurement consisted in the adsorption of the gas molecules on the surface of the material that occurs due to the interactions at the gas-solid interface. The analyzer registered the volume of gas adsorbed in the pore space of the rock. Knowing the sorption equilibrium points under a given pressure, it was possible to determine the structural parameters of the material.

In order to characterize the pores that get filled in with multiple layers the BET model was used [52] in the relative pressure range of 0.05 < *p*/*p*_0_ ≤ 0.30. It is based on the sorption isotherm that was plotted based on adsorption points according to Equation (1):

(1) $$a_{BET}\left(P\right)=\frac{a_{mBET}C\frac{p}{p_{0}}}{\left(1-\frac{p}{p_{0}}\right)\left[1+\left(C-1\right)\frac{p}{p_{0}}\right]},$$

where: $a_{BET}\left(P\right)$ (mmol/g) is the sorption equilibrium point, $a_{mBET}\;$(mmol/g) is the multilayer capacity, C (−) is the adsorption equilibrium constant that depends on the difference between the adsorption heat for the first layer and the condensation heat, p (Pa) is the absolute pressure, $p_{0}$ (Pa) is the saturation pressure.

This model assumes that the surface of the material is covered with multilayer of the gas molecules, the specific surface area of the adsorbed gas is determined by Equation (2):

(2) $$SSA=a_{m}\omega N_{A},$$

where: SSA (m^2^/g) is the specific surface area, $\omega \;\left({\mathrm{nm}}^{2}\right)$ is the surface occupied by one adsorbate molecule in the mono-molecular layer, $N_{A}\;$(1/mol) is the Avogadro’s number.

Barrett’s, Joyner’s and Halenda’s (BJH) theory [53] in the relative pressure range 0.01 < *p*/*p*_0_ was used to characterize the mesopores area of the material in which capillary condensation occurs. The BJH model is based on the Kelvin Equation (3) [54]. Based on sorption and desorption points, the total pore volume available for adsorbed gas particles, specific surface area and pore size distribution as a function of their diameter and mean pore diameter were determined:

(3) $$ln\left(\frac{p}{p_{0}}\right)=-\left(\frac{2\sigma V\cos\theta }{RTr_{k}}\right),$$

where: $\sigma \;(m^{3}/\mathrm{mol})$ is surface tension of liquid nitrogen, V (mol) is liquid molar volume of nitrogen, R (J/mol·K) is a gas constant, T (K) is temperature, $r_{k}\;\left(m\right)$ is capillary radius.

To characterize the volume and surface area of micropores in halloysites, t-plot and NLDFT models were used. The calculations of the t-plot method were made based on the results of the BET model and Harkins and Jura thickness equation in the thickness range of 0.35–0.5 nm. NLDFT analyzes were performed for pillared clays.

The macropores and a part of mesopores of the halloysites were analysed by mercury intrusion porosimetry (MIP). Measurements of the volume of mercury intruded into the open pores of the material were carried out on an AutoPore IV 9500 analyser (Micromeritics). Prior to the measurement the sample was heated at 378 K for 25 h and degassed under 6.5 Pa for 1h. The calculations are based on the Washburn theory [55]. The size of the critical capillary into which mercury can intrude under a given pressure is expressed by Equation (4). The surface area was determined from Equation (6) and the pore size distribution from Equation (7). As usual, in simplified calculations pressure p_0_ was omitted and constant value of mercury surface tension (0.48 N/m) and a constant mercury contact angle (141.3°) were adopted:

(4) $$D=-\frac{4\gamma cos\theta }{\Delta p},$$

assuming that:

(5) $$\Delta p=p_{1}-p_{2},$$

(6) $$S=-\frac{1}{\gamma \;cos\theta }\int_{0}^{V}pdV,$$

(7) $$f=-\frac{d(V)}{d\left(logD\right)}.$$

where: D (μm) is the critical pore diameter, γ (N/m) is the surface tension of mercury, θ (°) is the contact angle of mercury, Δp (Pa) is the difference of mercury pressure and gas pressure in the pores, $p_{1}\;\left(\mathrm{Pa}\right)$ is the hydrostatic pressure of mercury, $p_{2}\;\left(\mathrm{Pa}\right)$ is the gas pressure in pores of the samples, *S* (m/g^2^) is the surface area, *V* (cm^3^/g) is the pore volume.

**Figure 1:**
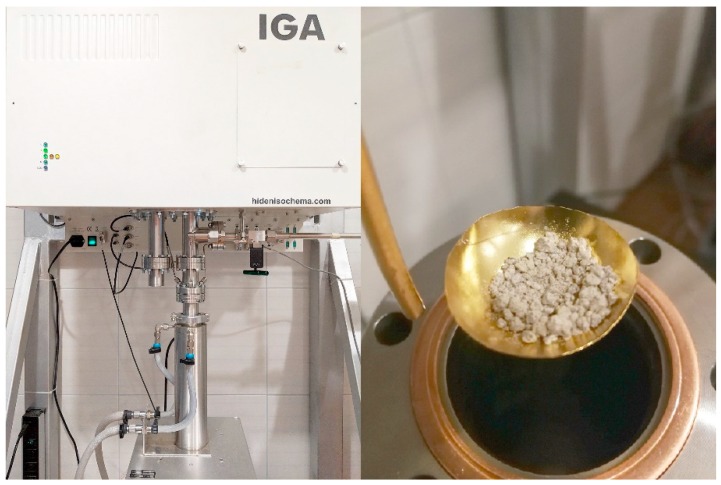
IGA 001 gravimetric gas sorption analyser.

The analyses of methane and carbon dioxide on halloysites were carried out using an IGA 001 gravimetric gas sorption analyser (Hiden Isochema, Warrington, UK), in the pressure range of 0–2.0 MPa (Figure 1). 

The gravimetric measurement method records the changes of the sorbent weight during sorption under isobaric and isothermal conditions. During diffusion the gas sorbate molecules permeate the pore structure of the sorbent. If the molecules get in the range of intermolecular interactions with the sorbent molecules the number of the degrees of freedom of the sorbate drops. This process is registered as an increase of the weight of the sorbate-sorbent system. Prior to the tests the samples were sieved. The 0.5–1.0 mm grain fracture was selected for the analysis and averaged using multiple cone splitting method. A 0.5 g sample was placed on the scale pan of the analyser in the reactor chamber. The measurement started with the sample preparation stage and then the sorption capacity of sample was determined to a selected gas at a given temperature and under a given pressure. Each time the procedure included:

- Sample preparation stage i.e., sieving a halloysite sample to the 0.5–1 mm grain fracture and degassing it in deep vacuum at 353 K for 12 h,
- The measurement stage i.e., the quasi-step change of the pressure of the gas sorbate in the reactor at the rate of 333 mbar/min and recording the changes of the weight of the sorbate-sorbent system for the period of time that it needs to equilibrate.

The measurements were carried out at 313 K and they were repeated for 0.1 MPa, 0.3 MPa, 0.6 MPa, 1.0 MPa and 1.5 MPa. Each time after changing the sorbate (CO_2_, CH_4_) a new sample was used to avoid changes in the sorption capacities resulting from previous tests. On the basis of the sorption points and by minimizing the sum of squared deviations Langmuir sorption isotherms were plotted according to Equation (8):

(8) $$a_{L}\left(P\right)=a_{mL}\frac{bp}{1+bp}+c,$$

where: $a_{L}\left(P\right)$ (mmol/g) is sorption equilibrium point, $a_{mL}$ (mmol/g) is the total monolayer capacity, b (1/MPa) is the adsorption equilibrium constant equal to the inverse of the half pressure, p (Pa) is the equilibrium pressure of the adsorbed nitrogen, c (mmol/g) is the coefficient representing the hysteresis loop.

In the range of the tested pressures, the Langmuir isotherms extrapolated the measurement points correctly in the case of both CO_2_ and CH_4_. From the point of view of the metrology of the phenomenon, the kinetics of sorption processes, understood as a decrease in the degrees of freedom of the sorbate molecules near the sorbent molecules is instantaneous. The slow achievement of sorption equilibrium results from the processes of molecule transport in the sorbent pore structure. The parameter that describes the sorption kinetics quantitatively is the effective diffusion coefficient. For the sphere with the radius R, Fick’s second law is:

(9) $$\frac{\partial c}{\partial t}=D_{e}\nabla^{2}C$$

but:

(10) $$D_{e}=\frac{D}{1+H}$$

takes the following form:

(11) $$\frac{\partial c}{\partial t}=D_{e}\frac{\partial^{2}c}{\partial r^{2}}$$

where: r is the distance from the sphere centre, C(r,t) is the distribution of the sorbate concentration within it. 

The solution to this equation has the form of a series expansion:

(12) $$\frac{M\left(t\right)}{M_{\infty }}=1-\frac{6}{\pi^{2}}\overset{\infty }{\underset{1}{\sum }}exp\left(-\frac{n^{2}\pi^{2}D_{e}t}{R^{2}}\right)$$

which describes how the mass M(t) of the substance accumulated in the spherical grain tends to a limit value $M_{\infty }$.

In order to determine the effective diffusion coefficient using a gravimetric method, it is necessary to record the changes of the weight of the sorbent-sorbate system as a function of time, after the step pressure change in the reactor. The maximum rate of the pressure changes in the case of IGA-001 is 333 mbar/min. One of the assumptions leading the analytical solution of the diffusion equation is the linear shape of the sorption isotherm. For the measurement uncertainties due to the isotherm being non-linear to be as small as possible, the test is usually made for the pressure change from vacuum to 0.1 MPa. 

## 3. Materials

The halloysite for testing came from the halloysite open pit mine in Dunino, Poland (51°08′44.0″ N 16°04′31.3″ E). Halloysite is an aluminosilicate belonging to the kaolinite-serpentine group with Al_2_Si_2_O_5_(OH)_4_ chemical formula. It occurs naturally in three variants of particle shapes: halloysite nanotubes, halloysite nanoplates and spheres. It consists of one octahedral aluminate sheet and one tetrahedral silicate sheet hence is classified as 1:1 phyllosilicate. Halloysite, like other phyllosilicates, is characterized by two-dimensional sheets consisting of SiO_2_ (tetrahedra) and AlO_3_ (octahedra). These two layers are separated by interlayer H_2_O molecules. Halloysite from polish deposit “Dunino” is a product of basalt weathering and a raw mineral in the deposit consists mostly of nanotubes and nanoplates. A detailed description of halloysite from Dunino deposit, its chemical and mineralogical characterization are presented in [56]. Table 2 shows the chemical composition and selected trace elements of raw halloysite determined by means of X-ray fluorescence method.

It occurs in two different hydration states; the dominant one is hydrated halloysite (10 Å) in which the water in the interlayer increases its thickness. It can also be found in dehydrated forms halloysite (7 Å). Occurs in two different hydration states, the dominant is the hydrated halloysite (10 Å), he is also found in dehydrated forms of halloysite (7 Å). In this work authors focused on the analysis of structural and sorption properties of dehydrated meta-halloysite and calcined halloysite.

For the purpose of the study, two samples were selected. Sample R-HAL was a raw halloysite which was initially dried at 378 K, which removed the interlayer water making it a meta-halloysite, the sample was not subjected to any further treatment except comminution for sorption experiments. The other sample (referred to as C-HAL sample) was a modified halloysite, calcinated at the temperature of 873 K.

**Figure 2:**
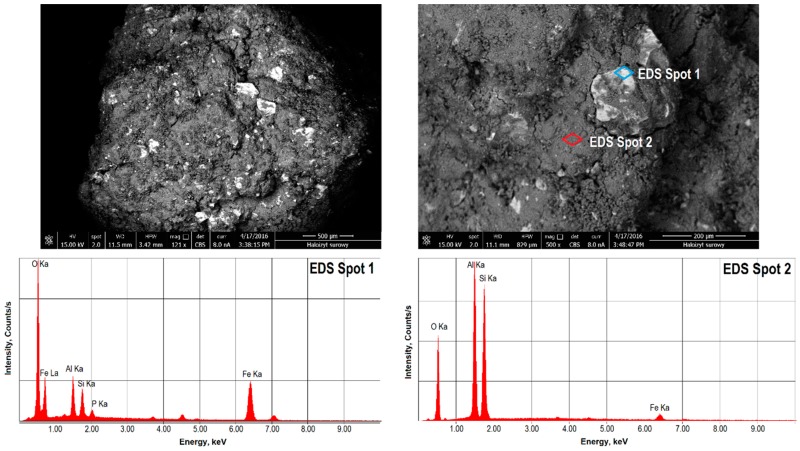
SEM image of the microstructure of the surface of raw halloysite R-HAL and chemical composition determined by an X-ray dispersion energy spectrometer (EDS).

**Figure 3:**
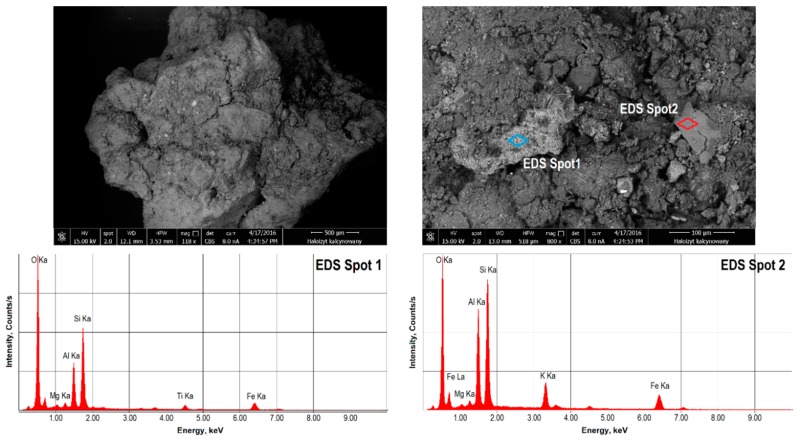
SEM image of the microstructure of the surface of calcined halloysite C-HAL and chemical composition determined by an X-ray dispersion energy spectrometer (EDS).

The SEM images (Figure 2 and Figure 3) show the microstructure of the raw Dunino halloysite and their phase diversity. Apart from the halloysite phase, the EDS analyses revealed a kaolinite phase and other mineral phases, which are irregular and differ in the surface microstructure. Those phases contained iron, magnesium and titanium compound, which may be indicative of the presence of magnetite, magnesium ferrite, ilmenite and geikielite [57].

**Figure 4:**
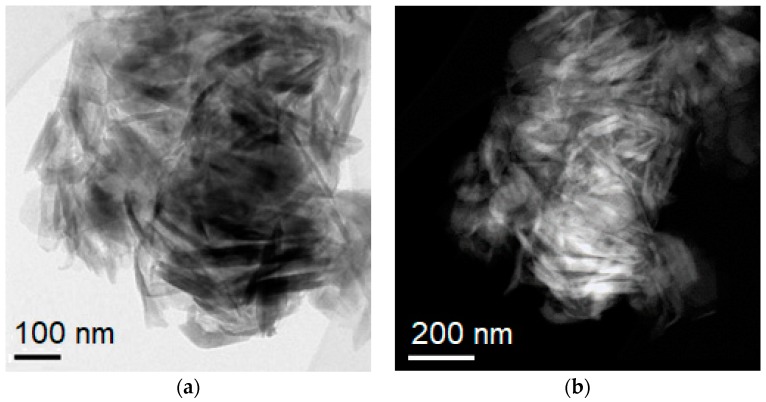
TEM images of raw halloysite samples showing halloysite nanoplates and nanotubes: (**a**) bright field (BF); (**b**) dark field (DF).

TEM images of halloysite samples (Figure 4) show the morphology of a raw Dunino halloysite. Grains consist of mixed halloysite nanoplates and halloysite nanotubes. Nanotubes are approximately 200–400 nm in length and have usually a thickness of 30–50 nm whereas nanoplates are characterized by an irregular shape and have usually a size of 80 nm in width and 400 nm in length.

## 4. Results

### 4.1. Structural Analyses

**Figure 5:**
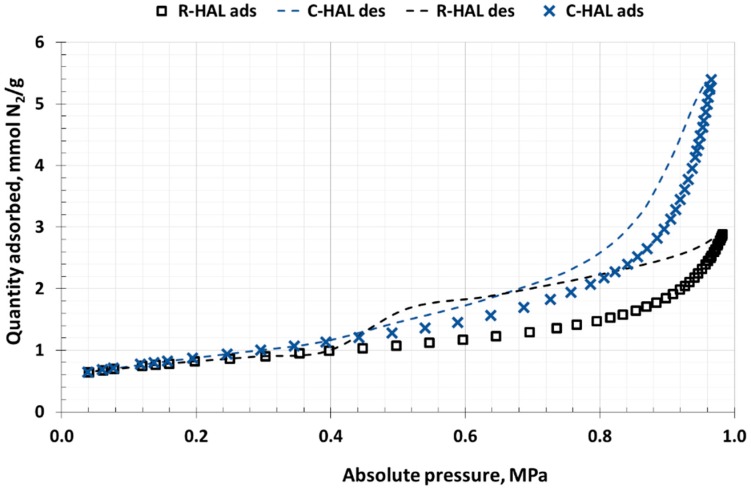
N_2_ sorption isotherms of halloysites R-HAL and C-HAL, 77K (LPNA method).

This LPNA and MIP methods were used to analyse the surface structures of the halloysite samples. Within LPNA analysis, nitrogen adsorption and desorption isotherms were plotted (77 K) (Figure 5) and micro and mesopore areas of the halloysites were described. In both samples type III isotherms were identified according to International Union of Pure and Applied Chemistry (IUPAC) [58]. Such an isotherm shape is typical of mesoporous and macroporous materials. In both samples capillary condensation of nitrogen took place in the multi-layer area. The desorption curve differed in shape from the adsorption curve creating in both cases a hysteresis loop of H3 type, characteristic of materials containing slit pores [59].

The isotherms were fitted based on the adsorption points as the function of pressure. The values of the structural parameters are presented in Table 3.

Assuming that the adsorbate molecules cover the surfaces of the tested materials completely, the BET multilayer surface area (*SSA_BET_*) as well as the BJH surface area (*SSA_BJH_*) were calculated. In the calculations it was assumed that the area covered by N_2_ molecules was ω = 0.162 nm^2^. In the raw halloysite the BET surface area was 62.7 m^2^/g, the BJH surface area was in the range of 53.9 m^2^/g and the total pores volume was 0.10 cm^3^/g. After calcination the values of the structural parameters increased. The value of *SSA_BET_* increased by nearly 70 m^2^/g, the value of *SSA_BJH_* was 72.5 m^2^/g and the *V_BJH_* increased to 0.18 cm^3^/g. To separate the area of micropores and mesopores, the t-plot method was used. According to this method, R-HAL had a negligible surface area of micropores (4.1 m^2^/g). After calcining, the surface of the micropores in C-HAL slightly developed to 16.3 m^2^/g. The obtained values of the structural parameters are at the level typical of macroporous carbonate rocks used in the energy industry [24,60,61].

**Figure 6:**
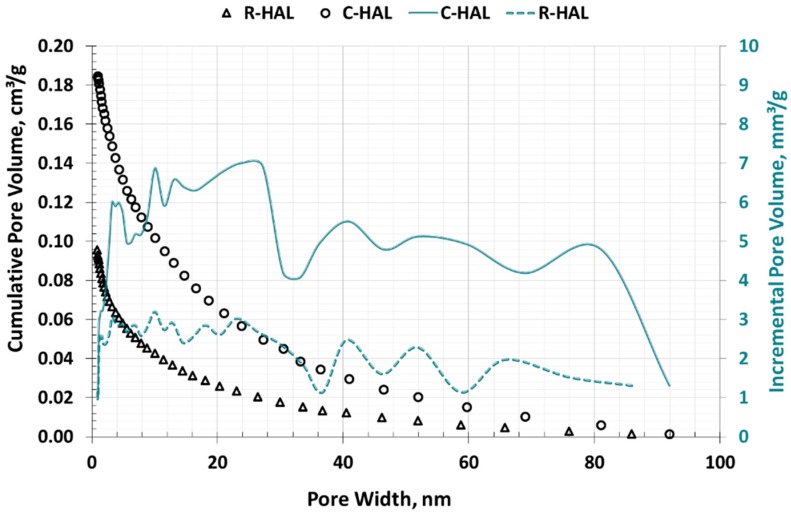
Distribution of pore volume as a function of their diameter in halloysites (LPNA method, BJH model).

Figure 6 presents the curves of the cumulative and incremental pore volume of halloysites as a function of their diameter, which was determined according to the BJH theory. This model assumes that the pores fill with gas on the principles of capillary condensation under the influence of increasing pressure. The C-HAL calcination sample had almost twice as large the cumulative volume in the mesopores area as the R-HAL non-calcined material.

**Figure 7:**
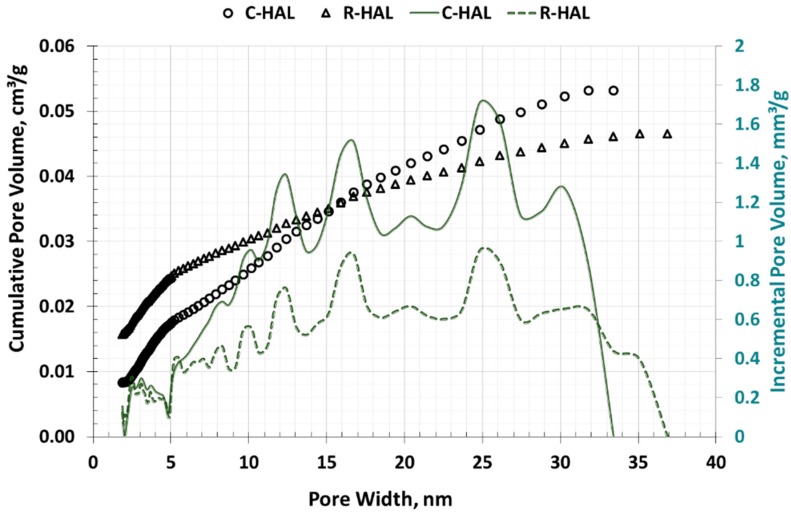
Pore size distribution in halloysites (LPNA method, NLDFT model).

Figure 7 presents the size distribution of mesopores in halloysites. Halloysite R-HAL had the largest pore volume with diameters in the range of 10–33 nm. After calcining (C-HAL), the value of mesopores volume almost doubled, and the pores with diameters from 12 nm to 32 nm had the largest volume.

**Figure 8:**
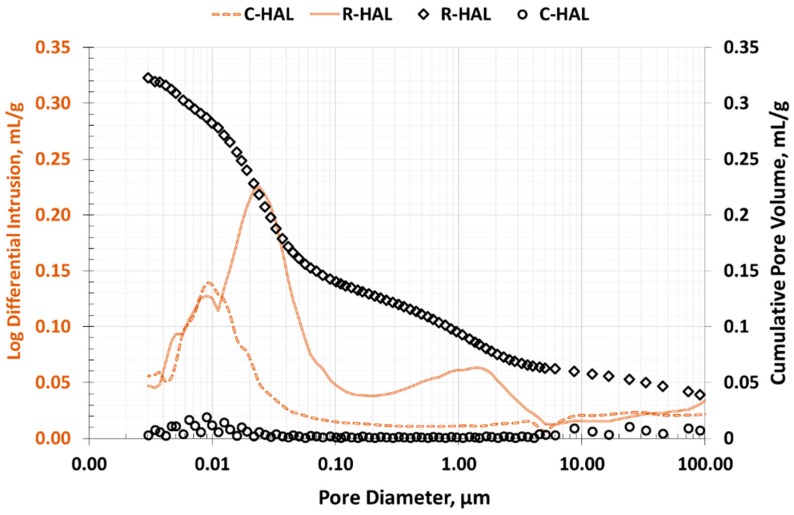
Distribution of pore volume as a function of their diameter in halloysite (MIP method) drawn.

To characterize the area of macropores in halloysite, they were subjected to mercury porosimetry (MIP) analyzes. The test results are summarized in Table 3. Halloysite showed diversification in the value of structural parameters but the halloysite after calcination had a much more extensive macroporous structure. The surface area in the C-HAL increased from 43 m^2^/g to 54 m^2^/g, while the porosity from 29% to 46%. Figure 8 shows the volume distribution of halloysite pores as a function of their diameter. In the R-HAL sample the pore distribution curve was unimodal, and the largest pore volume was in pores with a diameter of about 0.005–0.02 cm^3^/g. In the C-HAL sample, two ranges of pore diameters with the largest volume were distinguished. Fine macropores of 0.005–0.5 µm in diameter prevailed and a second pore peak with diameters of several µm was distinguished. 

### 4.2. CH_4_ and CO_2_ Sorption Analysis

On the basis of sorption capacities, registered under specified measurement conditions, CH_4_ and CO_2_ sorption isotherms were determined for halloysite samples. Table 4 presents the values of equilibrium sorption points at pressures 0.1, 0.3, 0.6, 1.0 and 1.5 MPa and the total sorption capacity relative to CH_4_ and CO_2_, approximated by Equation (8).

**Figure 9:**
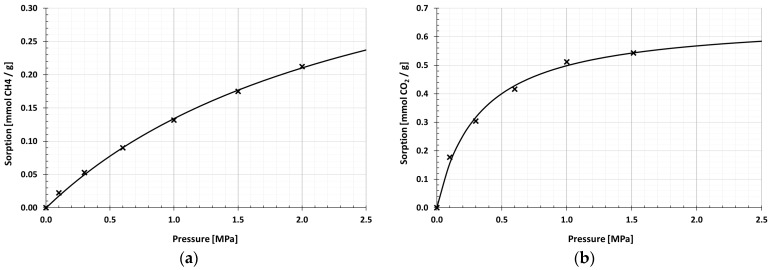
Sorption isotherms of raw halloysite: (**a**) CH_4_ sorption isotherm; (**b**) CO_2_ sorption isotherm.

**Figure 10:**
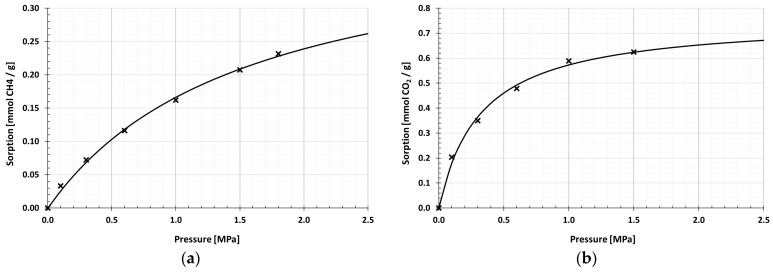
Sorption isotherms of calcined halloysite: (**a**) CH_4_ sorption isotherm; (**b**) CO_2_ sorption isotherm.

In the range of tested methane pressures, the sorption isotherms determined had a small course, well described by the Langmuir isotherm (Figure 9 and Figure 10). After methane sorption, the C-HAL calcination sample obtained higher values of sorption capacities at each pressure. The analysis of sorption points indicated that as the measurement pressure increased, so did the difference between CH_4_ sorption efficiency by R-HAL and C-HAL. The value of the maximum sorption capacity approximated by Equation (8) was in the range of 0.39–0.49 mmol/g. The obtained level of methane sorption capacity differed from the level typical for microporous materials, e.g., coal [12]. 

At the pressure of 0–1.5 MPa Langmuir sorption isotherms correctly extrapolated the measuring points also in the case of CO_2_. The carbon dioxide sorption value for both samples was over twice as high as for methane sorption. The value of the maximum sorption capacity determined by the Langmuir Equation (8) in the halloysite before calcining was 0.66 mmol/g. After the calcining process, it increased slightly to 0.76 mmol/g.

### 4.3. Diffusion Kinetics

**Figure 11:**
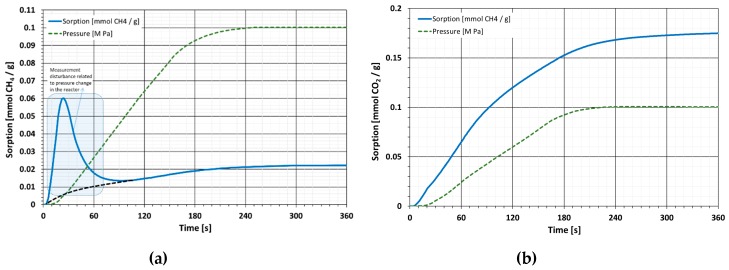
Diffusion kinetics of raw halloysite: (**a**) diffusion kinetic of CH_4_; (**b**) diffusion kinetic of CO_2_.

**Figure 12:**
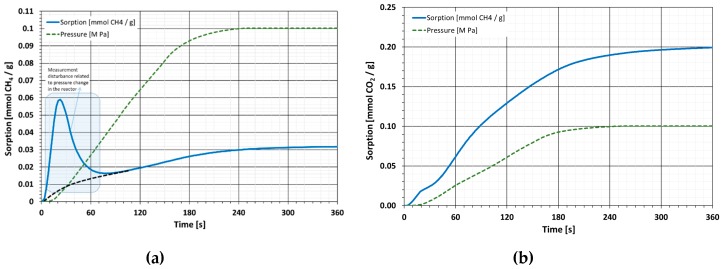
Diffusion kinetics of C-HAL calcined halloysite: (**a**) diffusion kinetic of CH_4_; (**b**) diffusion kinetic of CO_2_.

The kinetics of halloysite diffusion is so high that it is impossible to accurately determine the value of the effective diffusion coefficient by the gravimetric method. Analysis of the kinetics plots (Figure 11 and Figure 12) shows that changes in the mass of the sorbent-sorbate system almost immediately map the changes in sorbate pressure in the reactor. In this case, only a bottom estimate of the diffusion coefficient is possible [62].

Timofejew proposed some simplification of the solution to the analytical diffusion equation. After solving the Equation (12) looking for a moment of time for which the gas mass is 50% of the initial mass:

(13) $$\frac{6}{\pi^{2}}\left(exp\left(-\frac{\pi^{2}D_{e}t}{R^{2}}\right)+\frac{1}{2^{2}}exp\left(-\frac{2^{2}\pi^{2}D_{e}t}{R^{2}}\right)+\frac{1}{3^{2}}exp\left(-\frac{3^{2}\pi^{2}D_{e}t}{R^{2}}\right)+\ldots .\right)=\frac{1}{2}$$

we will obtain a solution described in the literature as Timofejew’s formula (14):

(14) De=0.308⋅R2π2⋅t12

where: t12 (s) is the half time, R (cm) is the so-called equilibrium radius of grain, equal:

(15) $$R=\frac{1}{2}\sqrt[{3}]{\frac{2\cdot d_{1}^{2}\cdot d_{2}^{2}}{d_{1}+d_{2}}}$$

The study used a grain size of 0.5–1mm, resulting in a radius R = 0.35 mm. In the case of CH_4_, both raw and calcined halves t12 can be estimated at about 90 s. This allows us to state that the value of the effective diffusion coefficient for CH_4_ on raw and calcined halloysite is higher than *De* > 4.2 × 10^−7^ cm^2^/s. In the case of CO_2_, the process is similar and the half time *t*_1⁄2_ can be estimated at about 120 s. With the same grain size, this means that the effective CO_2_ diffusion coefficient value for raw and calcined halloysite is higher than *De* > 3.1 × 10^−7^ cm^2^/s. The values of effective diffusion coefficients for halloysite are to be considered high. The comparison of these values with one of the materials most often tested for diffusivity such as coal, indicates that in the case of halloysite, the diffusion faster by 2 to 3 orders of magnitude.

## 5. Discussion

The use of porous materials in sorption processes depends on a number of properties. One of the decisive factors is a high sorption capacity. It is directly correlated with the high surface area (*SSA*) of the sorbent, the diameter of its pores and the chemical nature of its surface. An extremely important factor determining the use of sorbent is favorable adsorption kinetics. Phenomena associated with the accumulation of gas adsorbate in the pores of the material include filtration, diffusion and specific sorption processes. Specific sorption is an almost instantaneous process. Filtration is slower, but it is much faster than diffusion, which is the longest process. Therefore, the analysis of the gas transport process in the pores is mainly based on the analysis of the phenomenon of diffusion kinetics [63]. Some of the adsorbents, especially heterogeneous, are characterized by high selectivity, which depends on the size of the pores, their shape, tortuosity and distribution. Selectivity is a particularly important factor in the case of adsorption from multi-component solutions. Important factors in the applicability of adsorbents are also their good mechanical properties and good stability and strength as well as low production costs.

### 5.1. Structural Analyses

The surface area parameter is directly related to the amount and size of pores in the material. The pores may have different dimensions, shapes and volumes, and may form different channel systems. Due to the size and the adsorption phenomena occurring in them, according to IUPAC [58], pores are divided into macropores with a diameter above 50 nm, mesopores with a diameter of 2–50 nm and micropores with a diameter below 2 nm. Macropores act as transport channels in the processes of gas diffusion and filtration. Mesopores, also called transient pores, play a significant part in the adsorption mechanism., micropores on the other hand, make a decisive contribution to the size of the surface area of the material.

The pore structure of the examined halloysites mainly consists of mesopores and macropores. This is showed by the shapes of type III adsorption isotherms (LPNA method) and the relatively low BET surface value, which was in the range of 62.7–69.8 m^2^/g. T-plot analysis revealed that in R-HAL the share of micropores is negligible (6.5%), while in C-HAL it is 23.4%. The studies on the pore volume distribution determined by the LPNA method (NLDFT model) showed that the value of the total pore volume in the halloysite after calcination increased from 0.046 cm^3^/g to 0.054 cm^3^/g. In both halloysites, the largest volume was found in pores 10–33 nm wide, which according to IUPAC are classified as mesopores. According to the BJH model, which does not assume the presence of micropores, but only mesopores in which capillary condensation occurs, the total pore volume almost doubled its value, and the largest volume contained mesopores and fine macropores.

Halloysite belongs to the group of layered silicates. Other minerals of this group, such as: saponite, montmorillonite or vermicult, usually have a surface of several dozen m^2^/g [64]. The values of structural parameters of halloysite and other layered silicates are therefore comparable. The calcination used in the examined halloysite allowed for the increase the share of micropores in the mineral and increased *SSA* by 12%. The micropores do not have a large volume, but they affect the development of the surface area. In the case of adsorption processes, this is a very important factor. 

Comparing halloysite to other natural meso- and macroporous materials such as carbonate rocks (limestones and dolomites), halloysite has a much larger surface area and pore volume [24,60,61,65]. The *SSA_BET_* value of these rocks often do not exceed about 20 m^2^/g, hence they are not considered as reservoirs for CO_2_ or CH_4_. In contrast to mesoporous silica gels, halloysites have significantly lower *SSA_BET_* values. Typical silica gels have the surface area in the range 500–800 m^2^/g [66].

In comparison with microporous rocks, e.g., hard coal, the *SSA_BET_* value of halloysites is also much higher. The *SSA_BET_* of coal is only a few m^2^/g. However, coal is a highly selective rock in relation to gases. Nitrogen is a gas with which carbon interacts so weakly that the particles of this gas are not able to penetrate into its smallest pores. The heterogeneity of coal and its properties, such as swelling, for example, make comparing its structural parameters with other rocks very difficult [50].

Synthetic homogeneous materials such as carbon nanotubes or graphene compounds have a much more developed surface area than halloysite, reaching even several thousand m^2^/g. They have a well-developed and ordered structure but their production is rather expensive [48,67].

### 5.2. Sorption Analysis and Diffusion Kinetics

Analyses of the examined halloysite consisted in determining the sorption capacity for CO_2_ and CH_4_ gases. Due to the mainly meso- and macroporous structure of halloysite, the sorption processes described in the article were considered as physical adsorption processes. 

In the high pressure range the tested halloysites were characterized by sorption capacities. Individual sorption points made it possible to plot Langmuir sorption isotherm. The CO_2_ sorption capacities of the raw halloysite were in the range of 0.18–0.54 mmol/g. Calcination increased this parameter to a value in the range of 0.20–0.63 mmol/g. 

The sorption capacities of halloysite to CO_2_ were much higher than the sorption capacity to CH_4_, particularly in the lower pressures area. Raw halloysite in the pressure range of 0.1–1.5 MPa showed a CH_4_ sorption capacity from 0.02 mmol/g to 0.18 mmol/g, and after calcination it increased to 0.03–0.21 mmol/g. Langmuir’s total sorption capacity relative to CH_4_ was about 3 times lower than relative to CO_2_. Calcination increased the total CO_2_ sorption capacity from 0.66 mmol/g to 0.76 mmol/g, while in the case of CH_4_ sorption a decrease from 0.49 mmol/g (R-HAL) to 0.39 mmol/g (C-HAL) was noted.

The process of calcining led to an increase of the sorption capacity by several percent for individual pressures relative to raw halloysite. It is worth noting that in the case of CH_4_ sorption, calcination significantly affected the value of the *b* coefficient of sorption isotherms. Thanks to that change the Langmuir isotherm describing CH_4_ sorption on the calcined halloysite tends to an asymptotic value at lower pressures relative to the CH_4_ isotherm on the raw halloysite. As a result, despite the fact that sorption capacities relative to CH_4_ at the sorption points tested were higher in calcined halloysite than in the raw one, the Langmuir total sorption capacity based on extrapolation of measurement points was lower. Table 1 presents sorption capacities of various materials of natural and synthetic origin. The CO_2_ and CH_4_ sorption capacities of halloysite are comparable in value to high-carbonated coal.

The tested material displayed a very interesting sorption kinetics course. The diffusion process was so fast that it was impossible to precisely specify the diffusion coefficient value. This is due to the fact that the maximum rate of change of gas pressure in the reactor is comparable to the time to reach the value of asymptotic sorption. It is only possible to estimate that the De value is not lower than *De* > 4.2 × 10^−7^ cm^2^/s for CH_4_ and not lower than *De* > 3.1 × 10^−7^ cm^2^/s for CO_2_. Such high values of diffusion coefficients are very beneficial in terms of potential sorbent applications for the uptake of tested gases. 

Sorption kinetics plots for halloysite compared to synthetic nanomaterials were instant. The surface available for gas molecules was sorbed very quickly. According to [47], stabilization of mass change for carbon nanotubes and graphene-based materials as a result of sorption of both CO_2_ and CH_4_ took place after a few minutes. For these materials, however, it was not possible to quantify the process kinetics. 

Compared to natural coals, the values of diffusion coefficients in halloysite are significantly higher, even by one or two orders of magnitude. Phenomena resulting from the lengthening of sorption processes are mainly associated with the transport of gas particles inside the network of cracks and sorbent pores. This factor is marginal in the case of mesoporous materials but it is of great importance in the case of microporous materials. The duration of the diffusion of gas particles towards the pore volume depends on the pores the particles penetrate. The smaller the pores, the longer the diffusion time. 

## 6. Conclusions

The article compares the structure and sorption properties of calcined halloysite to CO_2_ and CH_4_ with the properties of raw halloysite. The research aimed to characterize halloysite for further structural modifications. The calcination process applied had an effect on the development of the pore space of the studied halloysite. Calcining at the temperature of 873 K led to opening of the smallest mesopores (up to 3.5 nm), but the values of both their volume and the surface area after calcination were very low. Calcining opened up the previously inaccessible macropores and increased the porosity of this clay rock by 17%. 

The CH_4_ and CO_2_ sorption properties of the measured halloysites was very interesting for technologies that require the use of significant amounts of sorbent. Compared to synthetic nanomaterials such as activated carbon, zeolites, modified graphene or nanotubes the sorption properties of halloysite are lower even by an order of magnitude. However, the cost of mining halloysite is much lower compared to the synthesis of nanomaterials. This is particularly true of geographical areas where this rock occurs in large quantities. It is due to that factor that the use of halloysite is beneficial.

Sorption capacities of halloysite are comparable to those of clay minerals and hard coal. Due to the equally high availability in nature, hard coal can also be a natural and cheap sorbent. However, low values of diffusion coefficients and low coal permeability pose a huge limitation in gas sorption on hard coal. These restrictions are a significant obstacle to the concept of CO_2_ storage in off-balance coal seams. Halloysites do not have this disadvantage because they have higher values of diffusion coefficients than typical hard coals by several orders of magnitude.

## Figures and Tables

**Table 1:** Literature review concerning sorption capacity of synthetic and natural sorbents.

Adsorbent	Temp.	Pressure	Sorption Capacity of CO_2_	Sorption Capacity of CH_4_	Ref.
-	K	MPa	mmol/g	mmol/g	-
**Activated Carbon—NCLK_3_, NCHA29, NORIT R2030CO_2_**	298–303	0.012	2.3–3.5	-	[39,40]
**Activated Carbon—CORK-DC**	298	0.01	1.8	-	[40]
**Biocarbon activated argon plasma**	278	0.1	-	1.21	[41]
		0.8	-	2.3	
		1.6	-	2.71	
**Zeolites—ZSM5, 13X**	298	0.012	0.7–4.5	-	[42]
**Zeolites—5A (Sinopec), Na-Beta**	298–303	0.01	2.7–4.55	-	[43,44]
**MOFs—Mg-Mof-74**	298	0.01	8.5	-	[45]
**MOFs—1C′-Li, 2C’-Li**	298	0.1	4.6–5.3	-	
**SWCNT funcionalized with zwitterions**	308	0–1.0	0.1	0.31	[46]
**SWCNT funcionalized with COOH ions**	308	0–1.0	3.2	6.21	
**MWCNT nanotubes**	278–283	0.1–1.0	0.11–0.6	0.07–0.3	[47]
	-	1.8–2.0	1.0	0.45	
	308–313	0.1–1.0	0.09–0.4	0.04–0.2	
	-	2.0	0.8	0.3	
	348-353	0.1–1.0	0.05–0.2	0.02–0.1	
	-	0.2	0.5	0.2	
**Highly porous activated graphene-derived carbon GDC from KOH activated TEGO**	298	0–3.5	21.1	11.3	[48]
**Reduced graphene oxide produced by modified Hummer’s method**	278–283	0.1–1.0	0.22–0.9	0.07–0.3	[47]
	-	1.5	1.0	0.4	
	308–313	0.1–1.0	0.18–0.8	0.06–0.3	
	-	1.5	0.9	0.3	
	348	0.1–1.0	0.1–0.5	0.05–0.2	
	-	1.5	0.6	0.3	
**Silicones with tin or platinum catalyst**	293	0.1	0.1	-	[49]
	-	1.5	1.0	-	
**Coal form Polish hard coal mines**	278–283	0.1–1.0	0.4–1.7	0.12–0.6	[12,16,47,50,51]
	-	1.6–2.0	1.1–2.3	0.6–1	
	308–313	0.1–1.0	0.12–1.2	0.06–0.6	
	-	1.6–2.0	1.0–1.7	0.5–0.9	
	348–353	0.1–1.0	0.2–0.8	0.07–0.4	
	-	1.6–2.0	0.7–1.2	0.4–0.6	
**Clay minerals—amino acid modified montmorillonite**	298	0.1	0.5	-	[36]

**Table 2:** Chemical composition and trace elements of halloysite (based on [56]).

Compound	wt%	Compound	wt%	Element	Concentration, ppm	Element	Concentration, ppm
SiO_2_	33.11	**TiO_2_**	2.23	**Zr**	180.9	**Sr**	96.3
Al_2_O_3_	28.87	**P_2_O_5_**	0.77	**Rb**	131.0	**Zn**	84.0
Fe_2_O_3_	17.92	**CaO**	0.71	**V**	124.9	**Cu**	73.8
LOI*	15.00	**MgO**	0.14	**Cr**	103.0	**Ni**	36.1

* LOI = loss on ignition.

**Table 3:** Structural parameters of halloysite (LPNA and MIP method).

**Low Pressure Nitrogen Adsorption—LPNA Results**		**R-HAL**	**C-HAL**
Total sorption capacity	mmol/g	0.627	0.712
BET specific surface area (*SSA_BET_*)	m²/g	62.7	69.8
t-plot micropore area	m^2^/g	4.1	16.3
t-plot external surface area	m^2^/g	58.6	53.5
BJH total pore volume (V_BJH_)	cm³/g	0.096	0.185
BJH surface area (*SSA_BJH_*)	m²/g	53.9	72.5
BJH average pore diameter	nm	7.10	10.19
**Mercury Intrusion Porosimetry—MIP Results**		**R-HAL**	**C-HAL**
Total Intrusion Volume	cm^3^/g	0.164	0.323
Total Pore Area	m^2^/g	43.48	54.04
Median Pore Diameter	um	0.02	0.05
Porosity	%	29.37	46.41
Permeability	mdarcy	0.01	1.5

**Table 4:** Sorption points and Langmuir halloysite isotherm parameters.

Sorbent	Sorbat	$a_{L}\left(0.1\right)$	$a_{L}\left(0.3\right)$	$a_{L}\left(0.6\right)$	$a_{L}\left(1.0\right)$	$a_{L}\left(1.5\right)$	$a_{mL}$	b
		mmol/g						1/MPa
R-HAL	CH_4_	0.02	0.05	0.09	0.13	0.18	0.49	0.38
	CO_2_	0.18	0.30	0.42	0.51	0.54	0.66	3.09
C-HAL	CH_4_	0.03	0.07	0.12	0.16	0.21	0.39	0.73
	CO_2_	0.20	0.35	0.48	0.59	0.63	0.76	3.08

where: $a_{L}\left(p\right)$ is the point of sorption equilibrium in pressure p. $a_{mL}$ is the total capacity of the monolayer, b is the Langmuir adsorption equilibrium constant equal to the inverse of half pressure.
